# Economic Burden of Depressive Symptoms Conditions among Middle-Aged and Elderly People with Hypertension in China

**DOI:** 10.3390/ijerph181910009

**Published:** 2021-09-23

**Authors:** Yun Wu, Dongbao Zhao, Jianwei Guo, Yingsi Lai, Lijin Chen, Sihui Jin, Yixiang Huang

**Affiliations:** 1 Department of Health Policy and Management, School of Public Health, Sun Yat-sen University, 74, Zhongshan 2nd Road, Guangzhou 510030, China; wuyun33@mail2.sysu.edu.cn (Y.W.); zhaodb3@mail2.sysu.edu.cn (D.Z.); guojw8@mail2.sysu.edu.cn (J.G.); chenlijin@mail.sysu.edu.cn (L.C.); jinsh7@mail.sysu.edu.cn (S.J.); 2 Department of Health Medical Statistics, School of Public Health, Sun Yat-sen University, 74, Zhongshan 2nd Road, Guangzhou 510030, China; laiys3@mail.sysu.edu.cn

**Keywords:** economic burden, depressive symptoms, hypertension, Chinese middle-aged and elderly

## Abstract

People with hypertension are more prone to incur depressive symptoms, while depressive symptoms have an obvious influence on the healthy functioning, treatment, and management of hypertensive patients. However, there have been limited studies on the association between depression and the economic burden of hypertension. We used data from the 2018 China Health and Retirement Longitudinal Study (CHARLS) to estimate the additional annual direct and indirect economic burden of depressive symptoms among middle-aged and elderly hypertensive patients with a multivariable regression model. The depressive symptoms were associated with substantial additional direct and indirect economic burden. Compared with non-co-MHDS (non-co-morbid hypertension and depressive symptoms) patients, the direct economic burden of lower co-MHDS (co-morbid hypertension and depressive symptoms) patients and higher co-MHDS patients increased 1887.4 CNY and 5508.4 CNY, respectively. For indirect economic burden, the lower co-MHDS patients increased 331.2 CNY and the higher co-MHDS patients increased 636.8 CNY. Both direct and indirect economic burden were incremental with the aggravation of depressive symptoms. The results showed depressive symptoms increased total healthcare costs by increasing the utilization and expenditure of primary healthcare services. Depressive symptoms also led to economic loss of productivity, especially for agricultural workers. This study highlights the importance of mental healthcare for hypertensive patients.

## 1. Introduction

The depression population has been increasing dramatically in recent years, with 258.2 million new cases reported worldwide in 2017 [1]. The burden of depression is expected to increase to 5.7% of the global disease burden by 2020 and major depression will rank as the first cause of disease burden worldwide by 2030 [2]. Hypertensive patients are more prone to suffer from depression. A meta-analysis study showed the prevalence of depression in people with hypertension was 26.8%, higher than that in the general population [3]. Additionally, several randomized controlled studies in China have demonstrated that hypertension is a risk factor for depression [4,5]. Hypertensive patients are vulnerable to psychological distress due to side effects of antihypertensive drugs, decreased quality of life, and health impairment [6]. Meanwhile, symptoms of depression are associated with higher odds of being hypertensive [7,8]. There are a great deal of studies indicating that depressive disorders have obvious impacts on hypertensive patients [9,10]. Depression and anxiety disorders can affect antihypertensive medication adherence and therapeutic effects [11,12]. It is pathophysiologically reasonable that depression affects hypertension, such as autonomic nervous system dysfunction [13]. In addition, depression significantly influences the blood pressure control of hypertensive patients [14]. Meanwhile, depression can indirectly affect the management of hypertension by inducing unhealthy behavioral factors, including smoking, excess alcohol use, and low levels of physical activity [15,16]. In addition, depression imposes a considerable health burden on hypertensive patients. Studies have shown that depressive symptoms have long-term negative effects on the physical and mental health of hypertensive patients, such as affecting self-management of hypertension and increasing the risk of functional disability, complications, and mortality [17,18]. The relationship between hypertension and depression can be described as cyclical, because symptoms of the latter may aggravate a patient’s health conditions which in turn leads to the increasing of distress or anxiety [19].

Mental health status affects the utilization of healthcare services and depression can cause higher healthcare costs of hypertensive patients at the macro level [20]. Studies from the US revealed that there were remarkable increases in total medical costs with increasing levels of treatment resistance in depression patients [21,22]. Studies have documented that depressive symptoms not only affect the health and well-being of people, but also lead to inevitable decrease in the work productivity [23]. A previous study based on 23 large American companies showed depressive symptoms were associated with higher disability rates, medical-related absent days, and total lost work days [24]. On the one hand, depression is associated with elevated direct and indirect costs of people. On the other hand, health impairments caused by hypertension also weaken people’s ability to work and increase health costs [25]. At present, the economic burden of depression and hypertension are both rising rapidly. In terms of depression, a study showed that the estimated annual direct and indirect expenditure of major depressive disorder increased from 173.2 billion dollars in 2005 to 210.5 billion dollars in 2010 in the United States [26].

In China, there were 245 million people affected by hypertension in 2019 and the number continues to increase [27]. As hypertensive patients co-morbid with depression are becoming more prevalent, a growing number of studies have focused on the association between depression and hypertension [28]. According to existing research, we know depression affects the treatment, physical function, and health outcomes of hypertensive patients [29]. However, little information is available on the economic effects of depression on hypertensive patients. A comprehensive assessment of the economic impacts of depression will highlight the importance of mental healthcare and improve the health service delivery model for hypertensive patients. Meanwhile, it will also provide valuable information for decision makers and medical workers on chronic disease management and public health interventions. Therefore, the objective of our study was to understand the effects of depressive symptoms on the utilization, expenditure of healthcare, and the loss of productivity in middle-aged and elderly people with hypertension.

## 2. Materials and Methods

### 2.1. Data Source

Data in our study were derived from the 2018 China Health and Retirement Longitudinal Study (CHARLS) investigation. CHARLS is a nationally representative longitudinal survey of Chinese people aged over 45 and their spouses, including information of demographic characteristics, health status, and economic circumstances of residents [30]. CHARLS adopted stratified (by per capital GDP of urban districts and rural counties) multi-stage (county/district/village/community/household) PPS (probability proportionate to size sampling) random sampling strategy. Respondents of CHARLS were followed every 2 years, using a face-to-face computer-assisted personal interview (CAPI). Our study used data from the fourth CHARLS survey, with 19,528 investigated individuals in total [31].

### 2.2. Study Sample

People identified as hypertensive patients included those diagnosed with hypertension by doctor, self-reported with hypertension, and found by CHARLS physical examination. The CHARLS physical examination adopted the criterion of systolic blood pressure (BP) ≥ 140 mmHg and/or diastolic BP ≥ 90 mmHg in defining hypertension [32]. Among 19,816 observations, 10,791 non-hypertensive observations and 2495 observations who refused to be interviewed or did not finish the survey were excluded. Finally, we included 6242 eligible individuals in our study, accounting for 32.0% of the total sample.

### 2.3. Heathcare Utilization and Expenditures

This study used healthcare utilization and expenditure to represent the direct economic burden of disease. The expenditure consisted of outpatient costs, inpatient costs, and self-medication costs, but not including non-medical costs (such as transportation, nursing, and accommodation costs) [33]. Self-medication costs referred to the expenses of self-seeking healthcare of hypertensive patients, mainly consisting of the cost of purchasing drugs at pharmacies [34]. The visit and cost of outpatient care and self-medication for hypertensive patients in a year were measured by the amount self-reported in the latest month, multiplied by 12. The inpatient visits and costs were calculated directly based on the self-reported amount in the past year [35]. Costs were presented in Chinese Yuan (CNY), the official conversion rate was 6.61 CNY per 1.00 USD in 2018.

### 2.4. Productivity Loss

In this study, human capital method was used to calculate the indirect economic burden of hypertensive patients. The indirect economic burden calculation was based on the economic loss of productivity associated with disability, including absence from one’s job and reduced ability to work. Unemployment lost days was defined as being unable to work because of illness or injury. Economic loss of unemployment was measured by average monthly wage income (¥2183.82 for urban residents/¥1234.28 for rural residents) multiplied by the number of unemployment months in a year. Missed work days were defined as the number of days that people missed work because of illness, injury, or emotional problems. Considering the gap both in the composition of occupation and wage income between rural and urban residents in China, we divided missed work days into agricultural lost days and employed lost days. Economic loss of missed work days was measured by the number of absent days in a year multiplied by the average daily wage income (¥104.82 for urban residents/¥59.25 for rural residents). We defined that there were 250 working days in a year. All the average monthly/daily wage incomes were based on the per capital wage income of (urban/rural) residents in 2012 released by the China National Bureau of Statistics, and then adjusted to 2018 levels by the Gross Domestic Product Index [36].

### 2.5. Criteria for Depression

Depressive symptoms were assessed by using the 10-item Center for Epidemiological Studies Depression Scale (CES-D-10), a simplified version of the depression self-rating scale. Previous research has reported the reliability coefficient (Cronbach’s coefficient) of these 10 indexes were above 0.76, which guaranteed the validation of CES-D-10 among middle-aged and elderly respondents in China [37]. CES-D-10 contains 8 negative questions and 2 positive questions, with answers on a four-scale metric, from rarely or none (<1 day), to not much (1–2 days), to sometimes or half of the time (3–4 days), to most of the time (5–7 days). The 4 options for the negative questions were assigned values from 0 to 3, while the positive questions were assigned values from 3 to 0. The total score ranges from 0 to 30, with a higher score representing that the depressive symptoms are more serious. We used universal criterion (score ≥ 10) to identify individuals with significant depressive symptoms [38,39]. We divided hypertensive patients into two groups based on the presence or absence of depressive symptoms, one group was defined as the co-morbid hypertension and depressive symptoms patients (co-MHDS patients), and the other group was defined as the non-co-morbid hypertension and depressive symptoms patients (non-co-MHDS patients). In order to observe the different economic burden effects of different depression degrees among hypertensive patients, our study divided depressive symptoms into two levels, defined as mild to moderate depressive symptoms (with a score of 10 ≤ X ≤ 20) and severe depressive symptoms (with a score of 21 ≤ X ≤ 30). As stated, the co-MHDS patients were divided into lower co-MHDS patients and higher co-MHDS patients.

### 2.6. Research Variables

In our study, the direct and indirect economic burden of middle-aged and elderly hypertensive patients were taken as outcome variables, including the healthcare utilization and expenditure, productivity missed days and relevant economic loss. For the selection of other covariates, we referred to social determinants and Anderson’s behavior model from the perspective of influencing healthcare utilization, including predisposing factors, enabling factors, and need factors [40]. Finally, we included gender, age, and marital status as predisposing factors. Enabling factors were comprised of rural–urban residents, education level, occupation, medical insurance kind, and household living expenditure. Need factors consisted of blood pressure control results, perceived health status, and comorbidity status. The comorbidity status was identified through the CHARLS investigation to find if the individuals have a chronic condition other than hypertension from a list of 11 conditions: dyslipidemia, diabetes, cancer, chronic lung disease, liver disease, heart disease, stroke, kidney disease, digestive disease, emotional or psychiatric problems, and memory-related disease [41].

### 2.7. Statistical Analysis

The statistical difference description of demographic characteristics between non-co-MHDS patients, lower co-MHDS patients, and higher co-MHDS patients were tested by Chi-square test. For multiple analysis, given that a large number of observations were found without any medical service utilization or productivity loss, we then compared the performance of zero-inflated Poisson regression and zero-inflated negative binomial regression models for further selection of the model. Finally, because of the nature of discreteness and overdispersion, both in healthcare visits and productivity lost days, we applied a zero-inflated negative binomial regression model to analyze outpatient visits, self-medication visits, and productivity lost days and we used a zero-inflated Poisson regression model to analyze the inpatient visits [42]. Considering the characteristics of consecutiveness and positive skewness in healthcare costs, we applied a generalized linear model (GLM) with gamma distribution and a log link to analyze that. Meanwhile, we applied GLM with negative binomial distribution and a log link on economic loss of productivity due to the characteristics of discreteness and positive skewness [43]. All estimated values used robust regression results controlled by key covariates including gender, age, marital status, Hukou, education level, health, and comorbidity status, to present the predictive margin effects. Statistical significance P value was set to 0.05, using two-tailed tests.

## 3. Results

### 3.1. Prevalence of Depressive Symptoms and Characteristics of the Study Population

The rate of depressive symptoms onset was 41.6% in middle-aged and elderly hypertensive people, with 34.2% in lower co-MHDS patients and 7.4% in higher co-MHDS patients. Compared with non-co-MHDS patients, co-MHDS patients were more likely to be female (P < 0.001), rural residents (P < 0.001), separated/divorced/unmarried status (P < 0.001), with no formal education (P < 0.001), unemployed (P < 0.001), having lower household living expenditure (P < 0.001), with new cooperative medical insurance (P < 0.001), in poor health (P < 0.001), and with more comorbidities (P < 0.001) (Table 1).

### 3.2. Estimating the Healthcare Utilization and Expenditure of Depressive Symptoms

The regression results showed that depressive symptoms were associated with higher healthcare use and cost among middle-aged and elderly people with hypertension. It was observed that the more serious the depressive symptom was, the heavier the medical burden was. Compared with non-co-MHDS patients, lower co-MHDS patients were shown to have suffered 1887.4 CNY additional cost for 2.0 additional healthcare visits in a year, and higher co-MHDS patients suffered 5508.4 CNY additional cost for 4.3 additional healthcare visits in a year (P < 0.05 for above). For outpatient services, the number of additional visits among higher co-MHDS patients was twice that of lower co-MHDS patients (1.4 vs. 2.8, P < 0.05 for both). The additional cost of higher co-MHDS patients reached 4305.0 CNY (P = 0.005). For inpatient services, the additional cost of lower co-MHDS patients was 1206.0 CNY (P = 0.003), while no significant higher cost was seen in co-MHDS patients. There were no significant additional inpatient visits in either of the two groups. For self-medication service, the amount of additional costs of higher co-MHDS patients was obviously higher than lower co-MHDS patients (394.0 CNY vs. 1019.8 CNY, P < 0.05 for both). The additional estimated values of outpatient costs in lower co-MHDS patients and inpatient costs in higher co-MHDS patients were not statistically significant (Table 2).

### 3.3. Estimating the Productivity Loss of Depressive Symptoms

The depressive symptoms were associated with additional productivity loss in middle-aged and elderly people with hypertension. Compared with non-co-MHDS patients, the entire annual incremental productivity economic loss of lower co-MHDS patients was 331.2 CNY for 5.4 additional missed work days, and for higher co-MHDS patients, it was 636.8 CNY for 11.9 additional missed work days (P < 0.05 for above). For unemployment, the number of additional lost days of higher co-MHDS patients was approximately twice as many as lower co-MHDS patients (2.8 vs. 5.7, P < 0.05 for both). The annual incremental economic loss of unemployment in lower co-MHDS patients appeared not to be statistically significant, while higher co-MHDS patients incurred 144.9 CNY for 5.7 additional missed work days (P < 0.05 for both). For agricultural lost days, the lower co-MHDS patients incurred 201.9 CNY due to 3.1 additional missed work days, while higher co-MHDS patients incurred 527.0 CNY due to an additional 7.2 missed work days (P < 0.05 for above). However, the additional employed lost days and employed economic productivity loss showed no statistical significance in both two groups. (Table 3)

## 4. Discussion

Our study found depressive symptoms were associated with additional direct economic burden on middle-aged and elderly hypertensive patients, and the economic loss was positively associated with depressive level. Compared with non-co-MHDS patients, the total additional direct economic burden of depressive symptoms reached 1887.4 CNY in lower co-MHDS patients and 5508.35 CNY in higher co-MHDS patients. The amount of additional direct economic burden was higher than the result of a previous study aimed at estimating incremental medical cost of depression among Chinese adults, which means the marginal direct economic burden of depression was more considerable in the middle-aged and elderly hypertensive patients [44]. On one hand, physical complaints from depression may drive hypertensive patients to go to a medical institution to seek healthcare, thus the utilization of psychotherapy and antidepressants will substantially increase the medical cost directly [45]. On the other hand, depression increased the healthcare costs indirectly by affecting the treatment and prognosis outcomes of hypertension [46]. A previous study has shown that both the diagnosis rate and treatment rate of depression in China were low [47]. When depressive symptoms are not able to be treated and alleviated in a timely manner, it tends to worsen the prognosis of the hypertension or other comorbidities. This will lead to corresponding higher medical expenditure, especially for those who are suffering severe depressive symptoms. Additionally, our study suggested that depressive symptoms increased the direct economic burden, mainly through increasing the utilization of outpatient and self-medication visits. We observed a 4305.0 CNY additional cost on outpatient care and 1019.8 CNY on self-medication in higher co-MHDS patients, which equaled 2.2-fold and 1.4-fold increases versus non-co-MHDS patients. This is roughly consistent with previous research based on hypertensive patients in the United States, except that our study did not find obvious incremental utilization of inpatient services [42].

Similarly to direct economic burden, we found depressive symptoms were also associated with increased additional indirect economic burden on middle-aged and elderly hypertensive patients, and the economic loss was mainly presented as agricultural productivity loss. On the basis of 324.8 CNY as the average agricultural productivity economic loss among non-co-MHDS patients, depression led to the increase in additional cost by 62.2% in lower co-MHDS patients and 162.3% in higher co-MHDS patients. The obvious incremental agricultural productivity loss may be associated with the higher rate of depressive symptom onset of rural hypertensive patients. Consistent with previous studies, our research results suggested the occurrence of depressive symptoms was strongly associated with rural–urban status. Compared with urban residents, rural residents faced more difficulties in accessing education, job opportunities, and better living environments, which can make rural hypertensive patients more prone to develop symptoms of anxiety or depression [48]. In addition, our study presented that employed people had higher risk of depressive symptom onset, but the results did not show that depressive symptoms would lead to additional economic lost for employed people. This was probably because Chinese employees were generally under work stress and unlikely to be absent from work. A previous foreign study examining the economic burden of adults with major depressive disorders found that the amount of productivity economic loss was larger than the expenditure of healthcare. However, our research results showed the opposite [26]. This might be related to the low employment rate and low wage level of the middle-aged and elderly population in China caused by weak knowledge structure, skills, and physical strength [49].

The increased economic burden of depressive symptoms highlights the importance of screening, treatment, and management of depression among middle-aged and elderly hypertensive patients. Implementing routine screening of depression among hypertensive patients and then referral for appropriate treatment can reduce the occurrence and development of depression in the early stage, thus reducing the risk of health damage and corresponding healthcare costs [50]. For treatment of hypertension, we should integrate physical healthcare and mental healthcare. High-income countries have proposed implementing collaborative depression care in guidelines, which has been demonstrated to be effective in clinical treatment and as a cost-saving measure [51]. In low-resource settings, including China and other developing countries, research has suggested that it is more cost-effective to implement primary-care-based collaborative care [52]. Our study indicated that depressive symptoms affected hypertensive patients mainly by increasing the demand for primary or secondary healthcare. Thus, we can set up mental health counseling services in community health institutions so that we can diagnose depression and provide patients timely primary care. As for management of depression in hypertensive patients, studies have shown that collaborative depression care management conducted by a physician team is effective [53]. High-income countries created a cooperative primary care provider team to provide comprehensive health services for chronic patients, including regular symptom monitoring, health education, and social support [54]. In China, diagnosis, treatment and long-term follow-up for chronic diseases are provided by primary healthcare institutions. The treatment of hypertension only includes antihypertensive drug use and lifestyle interventions [55], therefore, it is essential to establish a multidisciplinary team dominated by general practitioners to manage the physical and mental health of hypertensive or other chronic patients. Meanwhile, primary health institutions should strengthen the cultivation of general practitioners on psychological diagnosis, treatment, and education [56].

Given the increasing healthcare needs of hypertensive patients, especially those living in rural areas with higher risk of depression but worse medical resources and economic conditions, governments should increase funding for primary care of mental health and add depression screening as well as psychological counseling into basic public health service packages. In addition, our study showed those who suffered more comorbidities were also prone to develop depressive symptoms and usually had serious health damage. So, medical institutions and practitioners should treat them as high-risk population for depression and provide them priority in health treatment and management.

Based on our results, it was estimated that the total economic burden of middle-aged and elderly Chinese co-MHDS patients was 1259.5 billion CNY in 2018, accounting for 1.4% of the GDP in the same year. The direct economic burden (medical expenditure) accounted for 21.3% of the total national health expenditure. As the aging process accelerates, the economic burden of co-MHDS patients will also put great pressure on healthcare funds. The government should improve the medical security level and compensation ratio of medical services related to the treatment of hypertension patients. Moreover, health service providers should pay more attention to screening and prevention in chronic patients, as well as mental health intervention and treatment.

There are several limitations in our study. Firstly, our study used data in a cross-sectional design that could not determine a causal relationship between hypertension and depressive symptoms. Follow-up studies and panel data analysis are needed in further studies. Secondly, our study used a retrospective approach to collect data, including healthcare visits, expenditure, and missed work days, which might generate information bias. When hypertension patients were not clear about how much they spend on health services, we recorded the range of cost values they answered. Given that our study subjects were middle-aged and elderly people, of which some had poor memory, influence of information bias might be more considerable.

## 5. Conclusions

Our study is the first to calculate productivity economic loss of depressive symptoms among hypertensive patients. It is also the first to prove that depressive symptoms are associated with an increase in economic burden in Chinese middle-aged and elderly people with hypertension. Depressive symptoms will increase overall healthcare costs through increasing use and expense of primary healthcare services and incur economic loss of productivity, especially for agricultural workers. Therefore, we should be aware of the importance of mental healthcare for hypertensive patients. Governments should develop action plans to promote mental health well-being and perfect the mental healthcare system. Health institutions and physicians should implement collaborative primary care in hypertension treatment and management. Future studies should focus on the effectiveness of incorporating mental healthcare in the treatment and management of hypertensive patients, including clinical efficacy and economic assessment.

## Figures and Tables

**Table 1:** Demographic characteristics of different depressive statuses among middle-aged and elderly people with hypertension.

Character Variables	Non-co-MHDS Patients N (%)	Lower co-MHDS Patients N (%)	Higher co-MHDS Patients N (%)	P
	n = 3648	n = 2134	n = 460	
Gender				
Male	1988(54.5)	893(41.9)	128(48.2)	<0.001
Female	1660(45.5)	1241(58.2)	332(72.2)	
Age				
45–54	643(17.6)	367(17.2)	67(14.6)	0.674
55–64	1207(33.1)	706(33.1)	146(31.8)	
65–74	1269(34.8)	747(35.0)	177(38.5)	
≥75	529(14.5)	413(14.7)	70(15.2)	
Marital status				
Married	3165(86.8)	1735(81.3)	333(72.4)	<0.001
Widowed	68(1.9)	49(2.3)	17(3.7)	
Separated/Divorced/Unmarried	415(11.40)	350(16.4)	110(23.9)	
Hukou				
Rural	2566(70.3)	1755(82.20)	394(85.7)	<0.001
Urban	1082(29.7)	379(17.7)	66(14.4)	
Education level				
No formal education	1344(36.9)	1074(50.3)	304(66.1)	<0.001
Primary education	851(23.3)	479(22.5)	92(20.0)	
Secondary education	865(23.7)	388(18.2)	51(11.1)	
High School and above	588(16.1)	193(9.0)	13(2.9)	
Occupation				
Retired/Receded	461(12.6)	157(7.4)	21(4.6)	<0.001
Unemployed	1192(32.7)	814(38.1)	221(48.0)	
Agricultural work	385(10.6)	144(6.8)	19(4.1)	
Employed	1358(37.2)	908(42.6)	178(38.7)	
Self-employed/Unpaid helper	252(6.9)	111(5.2)	21(4.6)	
Medical insurance				
Urban employee	732(20.1)	220(10.3)	22(4.8)	<0.001
New cooperative	2016(55.3)	1435(67.2)	339(73.7)	
Urban and rural resident	500(13.7)	277(13.0)	52(11.3)	
Urban resident and others	330(9.1)	137(6.4)	28(6.1)	
No insurance	70(1.9)	65(3.1)	19(4.1)	
Household living expenditure				
Lower 50%	1908(52.3)	1337(62.7)	319(69.4)	<0.001
Higher 50%	1740(47.7)	797(37.4)	141(30.7)	
Perceived health status				
Poor	780(21.4)	1037(48.6)	335(72.8)	<0.001
Fair	2034(55.8)	900(42.2)	109(23.7)	
Good	834(22.9)	197(9.2)	13(3.5)	
Comorbidity status				
No comorbidity	988(27.1)	313(14.7)	32(7.0)	<0.001
1 comorbidity	1084(29.7)	569(26.7)	98(21.3)	
2 comorbidities	769(21.1)	498(23.3)	97(21.1)	
≥3 comorbidities	807(22.1)	754(35.3)	233(50.7)	
Blood pressure results				
Have controlled	3146(86.2)	1650(77.3)	328(71.3)	<0.001
Not controlled	502(13.8)	484(22.7)	132(28.7)	

**Table 2:** Additional healthcare utilization and expenditure of depressive symptoms among middle-aged and elderly people with hypertension.

	Lower co-MHDS Patients		Higher co-MHDS Patients	
	Marginal Effects	95%CI	Marginal Effects	95%CI
Healthcare utilization				
Total visits	2.0 ***	1.0–3.0	4.3 ***	2.4–6.3
Outpatient	1.4 **	0.6–2.3	2.8 **	1.1–4.5
Inpatient	0.1 **	0.0–0.1 a	0.1 **	0.0–0.2 b
Self-medication	0.5 **	0.2–0.8	0.8 **	0.4–1.3
Healthcare expenditure				
Total cost	1887.4 **	381.2–3393.6	5508.4 **	2010.7–9006.1
Outpatient	380.5	−510.3–1271.2	4305.0 **	1275.1–7334.8
Inpatient	1206.0 **	412.2–1999.8	544.2	−193.2–1281.6
Self-medication	394.0 **	71.0–716.9	1019.8 **	417.1–1622.4

Note: (1) Robust regression adjusted by gender, age, marital status, Hukou, education level, health, and comorbidity status were used to get the results in all models. (2) Study groups were based on the score of CES-D-10: non-co-MHDS patients (score < 10), lower co-MHDS patients (score 10 ≤ X ≤ 20), higher co-MHDS patients (score 21 ≤ X ≤ 30). (3) ** *p* < 0.05, *** *p* < 0.01. (4) a: 0.009–0.083; b: 0.024–0.154.

**Table 3:** Additional productivity days and economic loss of depressive symptoms among middle-aged and elderly people with hypertension.

	Lower co-MHDS Patients		Higher co-MHDS Patients	
	Marginal Effects	95%CI	Marginal Effects	95%CI
Days of productivity lost				
Total days lost	5.4 **	1.4–9.4	11.9 **	3.5–20.2
Unemployment	2.8 *	−0.3–5.7	5.7 **	0.2–5.7
Agricultural	3.1 ***	1.8–4.4	7.2 ***	3.9–10.6
Employed	0.2	−0.3–0.7	0.3	−0.2–0.9
Economic loss of productivity				
Total economic lost	331.2 **	118.0–544.4	636.8 ***	279.0–994.7
Unemployment	39.6	−18.9–98.3	144.9 **	32.6–257.2
Agricultural	201.9 ***	103.7–300.1	527.0 ***	283.3–770.6
Employed	−0.2	−0.5–0.1	0.4	−0.6–1.3

Note: (1) Robust regression adjusted by gender, age, marital status, Hukou, education level, health, and comorbidity status were used to get the results in all models. (2) Study groups were based on the score of CES-D-10: non-co-MHDS patients(score <10), lower co-MHDS patients (score 10 ≤ X ≤ 20), higher co-MHDS patients (score 21 ≤ X ≤ 30). (3) * *p* < 0.1, ** *p* < 0.05, *** *p* < 0.01.

## Data Availability

The datasets generated and/or analyzed during the current study are available in the CHARLS repository, http://charls.pku.edu.cn/index/en.html.
